# Communication Patient Reported Outcome Measures for Adults With Communication Disorders: A Systematic Review of Content Validity

**DOI:** 10.1111/1460-6984.70050

**Published:** 2025-05-12

**Authors:** Lizet Van Ewijk, Katerina Hilari, Analisa Pais, Anna Volkmer

**Affiliations:** ^1^ Research Group Speech and Language Therapy, Participation through Communication, Research Centre Health and Sustainable Living HU University of Applied Science Utrecht the Netherlands; ^2^ Centre for Language and Communication Science Research, School of Health and Psychological Sciences, City University of London London UK; ^3^ School of Health and Social Care University of Essex Colchester UK; ^4^ Division of Psychology and Language Sciences University College London London UK

**Keywords:** communication, communication disorders in adults, content validity, outcome measure, PROM, psychometrics

## Abstract

**Background:**

Content validity is a key measurement property that should be considered when selecting or reviewing a patient‐reported outcome measure (PROM). In the field of communication disorders, there are several PROMs available, most of which are disease specific. It is unknown what the quality of the content validity of these PROMs is.

**Aims:**

This study aimed to evaluate the content validity of existing communication PROMs used with adults with communication disorders.

**Methods:**

This study evaluated PROMs drawn from a previously published systematic literature review. Of 31 measures, 25 measures were included in this review, covering a range of communication‐related constructs in different communication disorders. The process of rating followed the COnsensus‐based Standards for the selection of health Measurement INstruments (COSMIN) methodology for assessing the content validity of PROMs. There were three stages to the evaluation process comprising Step 1: evaluating the quality of the PROM development, Step 2: evaluating the quality of content validity studies on the PROM (if available) and Step 3: evaluating the content validity of the PROM overall, based on the quality and results of the available studies and the PROM itself.

**Main Contribution:**

Step 1: With regards to the quality of the PROM development, 21 of 25 PROMs were rated as inadequate. Step 2: Content validity studies were available for five of the PROMs. All of these studies were rated doubtful or inadequate. Step 3: The quality of the available evidence on content validity of the included PROMs was overall very low. Only the evidence on the content validity of the Communication Participation Item Bank (CPIB) and the Neuro‐QoL (Quality of Life in Neurological Disorders) was rated as of moderate quality.

**Conclusions:**

Results of this study highlight the scarcity of high‐quality evidence on the development and content validity of PROMs that aim to capture the construct of communication. This review is a call to action for future PROMs to include both the target population and professionals in development and content validity testing, using rigorous methodology in the process.

**WHAT THIS PAPER ADDS:**

*What is already known on this subject*

- There are several patient reported outcome measures (PROMs) available for adult communication disorders. Many of these PROMs have been assessed on one or more psychometric properties, typically reliability and validity aspects. Content validity is often overlooked in research.

*What this study adds*

- Clinimetric and psychometric experts recommend content validity as the first and most important measurement property to consider when selecting a scale. This study is the first to provide a systematic assessment of the quality of content validity of communication PROMs used in adult communication disorders.

*What are the potential or clinical implications of this work?*

- Using measures with good content validity during outcome measurement ensures that researchers and clinicians capture constructs that are relevant and important to clients. It also ensures that the measures used are accessible to the clients and comprehensively address their needs.

## 1 Introduction

Patient‐reported outcome measures (PROMs) are scales that collect information about health outcomes directly from individuals who are experiencing a particular condition or receiving a specific treatment. PROMs provide a way to capture the patient's perspective on their symptoms, functional status, quality of life, and other relevant aspects of their health (Churruca et al. 2021). With the increased emphasis in recent years on person‐centred care (Coyne et al. 2018), PROMs are gaining prominence in healthcare. They provide a rigorous approach to capturing essential evidence that is otherwise challenging to obtain in a formal manner. According to de Riesthal and Ross (2015, 116), ‘the patient is the exclusive source of data for certain therapeutic effects’, highlighting the need to actively solicit information that meaningfully integrates the client's subjective experience and preferences. PROMs offer a standardized and quantifiable method for measuring key components of evidence‐based practice (EBP) (Sackett et al., 1996; Cohen and Hula, 2020).

Guidance on how to assess the psychometric properties of PROMs and how important these properties are for the reliability and validity of measurement was developed in 2010. (Mokkink et al., 2010) The international Delphi COSMIN study (COnsensus‐based Standards for the selection of health status Measurement INstruments) informed the development of a consensus‐based checklist to evaluate the methodological quality of PROMs and studies on measurement properties.

The first area on the COSMIN checklist (Mokkink et al., 2018) concerns content validity. Content validity is ‘the degree to which the content of a scale is an adequate reflection of the construct to be measured. It refers to the relevance, comprehensiveness, and comprehensibility of the PROM for the construct target population, and context of use of interest’ (Terwee, Prinsen, Chiarotto, Westerman, et al. 2018, 1159). It is considered the most important measurement property of a PROM; lack of content validity affects all other measurement properties. As Terwee et al. describe, irrelevant items may decrease internal consistency, structural validity, and interpretability of the PROM. A high Cronbach's alpha is no guarantee that the construct of interest is being measured or that no important concepts are missing, and a high test–retest reliability or responsiveness does not imply that all items are relevant or that no important concepts are missing.

Content validity has also proven to be the most challenging to assess (Terwee, Prinsen, Chiarotto, Westerman, et al. 2018). Assessment involves gathering feedback from both clients and professionals regarding the relevance, comprehensiveness, and comprehensibility of various aspects such as a PROM's items, response options, and instructions. In 2018, Terwee and colleagues conducted an international Delphi study to establish criteria for what constitutes ‘good’ content validity. This resulted in a detailed, standardised guideline for the assessment of content validity in PROMs. Ten criteria for good content validity were defined regarding item relevance, appropriateness of response options and recall period, comprehensiveness, and comprehensibility of the PROM (Terwee, Prinsen, Chiarotto, de Vet, et al. 2018).

In the context of communication disorders, PROMs allow for the assessment of the impact of these difficulties on individuals' daily functioning, social interactions, and overall well‐being. Communication disorders can significantly affect an individual's ability to communicate effectively, which can lead to various psychosocial, emotional, and functional challenges (cf. Beilby et al. 2013 for stammering, or Lam and Wodchis 2010 for aphasia). PROMs provide clinicians and researchers valuable insights into the patient's subjective experiences, allowing for a comprehensive understanding of the impact of these disorders on their lives.

There are several PROMs available for adult communication disorders, most of which are disease specific. Examples include the SAQOL‐39 g (Hilari et al. 2009), which captures quality of life from the perspective of the client after stroke, or the Voice Handicap Index (Rosen et al. 2004), which aims to capture the impact of a voice problem on the client's daily life. Many of these scales have been assessed on one or more psychometric properties, often reliability and validity aspects, such as internal consistency, test–retest reliability, interrater reliability, or structural validity.

More specifically in the area of communicative functioning, Eadie et al. (2006) undertook a search for relevant PROMs related to this construct. They highlight that, in the context of the International Classification of Functioning, Disability and Health (ICF; WHO 2001), there are numerous available scales that address body functions and structures, but a scarcity of instruments, as performance challenges, shifts away from being predominantly biomedical in nature. Embracing the ICF as a framework, they suggest that speech and language therapists (SLTs) should assess communication within social contexts, with an emphasis on ‘*communicative participation*’. The overall purpose of their article was ‘to review self‐reported instruments of ***communicative functioning*** to determine how well we are addressing the participation component of the ICF model in the field of speech‐language pathology’ (Eadie et al. 2006, 2). Their review was focused at the level of the individual items of the six scales found, not the validity of the scales themselves. Their review led to the development of the Communicative Participation Item Bank (CPIB) (Yorkston et al. 2008).

In 2023, Ter Wal et al. updated this work, with the purpose of expanding the CPIB item pool. The review followed the COSMIN guideline for systematic reviews (Prinsen et al. 2018). In line with the original review by Eadie et al., their search aimed to find PROMs designed to measure communication in an adult population. A systematic search of several databases was undertaken from January 2006 (where Eadie et al. 2006; left off) to December 2021. Abstracts were included if: (1) They described a scale aiming to measure communication, including scales that aim to measure the impact of communication difficulties on daily life, or quality of life. (2) The scale described was a PROM (i.e., self‐reported) that had to be completed by an adult with communication difficulties. (3) There was information available on the measurement properties (i.e., on the development, validity, reliability, or other measurement properties). (4) The article was written in English or Dutch. A full description of the search strategy, including a complete set of search terms and inclusion/exclusion criteria used to search for the PROMs, are reported in Ter Wal et al. (2023) and reproduced in Supporting Information S1 with permission. Thirty‐one scales were initially identified for inclusion in that study. As in the original review by Eadie et al. (2006), the scales themselves were not reviewed.

Given the increased knowledge on and interest in PROM development and particularly the recent developments in methodological knowledge on content validity and its importance, a thorough review of the content validity of these PROMs seemed of value. The purpose of the current study was therefore to assess the content validity of PROMs that aim to capture communication and were developed for people with communication (speech, language, voice, or hearing) difficulties. This work is an extension of the Ter Wal et al. (2023) review and addresses the research question: What is the content validity of existing PROMs used with adults with communication disorders, according to the COSMIN guidelines?

## 2 Materials and Methods

This study evaluated PROMs drawn from the Ter Wal et al. (2023) systematic review. From these (*n* = 31), 25 measures were included, covering a range of communication‐related constructs in different communication disorders. These are detailed in Table 1. Six measures were excluded: tinnitus measures (*n* = 3) were excluded as tinnitus was not considered a communication disorder. A further three scales were excluded: the Communication and Language Assessment Questionnaire for People with Multiple Sclerosis (CLAMS) as Ter Wal et al. (2023) found that 0 items were relevant to the construct of communicative participation; the Communication Disability Profile (CDP) was excluded as a unique scale but instead included as part of the development of the Aphasia Impact Questionnaire (AIQ). Lastly, the Stroke Communication Scale (SCS) was excluded, as it is only available in Portuguese. Although a direct translation exists, for the review of content validity, the reviewers must be able to rate the comprehensibility of the original (or cross‐linguistically adapted) items and none of the reviewers spoke Portuguese.

**TABLE 1:** Description of the 25 measures included in this review.

PROM	Reference to first article	Construct(s)	Target population	(Sub)scale (s) (number of items)	Response options	Original language
**Aphasia Communication Outcome Measure (ACOM)**	Doyle, P. J., M. R. McNeil, K. Le, W. D. Hula, and M. B. Ventura. 2008. “Measuring Communicative Functioning in Community‐Dwelling Stroke Survivors: Conceptual Foundation and Item Development.” *Aphasiology* 22, no. 7–8: 718–728.	Communication functioning	People with aphasia	Three versions: full 59‐item version, a 12‐item computer adaptive version, and a 12‐item static short form. Covers talking, comprehension, writing, and naming.	Vertical rating scale with four categories: not at all, somewhat, mostly, and completely, and showing the boundaries between the categories at 30%, 70%, and 99%.	English
**Aphasia Impact Questionnaire (AIQ)**	Swinburn, K., W. Best, S. Beeke, M. Cruice, L. Smith, E. Pearce Willis, K. Ledingham, J. Sweeney, and J. S. McVicker. 2019. “A Concise Patient Reported Outcome Measure for People With Aphasia: The Aphasia Impact Questionnaire 21,” *Aphasiology* 33, no. 9: 1035–1060.	Impact of aphasia/ multiple domains	People with aphasia	21 items, 3 subscales: 1. Activities, 2. Participation, 3. Emotional state/well‐being	Rating scale with pictures and descriptors, scored 0–4. Different versions for different sexes/race.	English
**Assessment of Language Use in Social Contexts for Adults (ALUSCA)**	Valente, A. R. S., A. Hall, H. Alvelos, M. Leahy, and L. M. T. Jesus. 2019. “Reliability and Validity Evidence of the Assessment of Language Use in Social Contexts for Adults (ALUSCA).” *Logopedics, Phoniatrics, Vocology* 44 no. 4: 166–177.	Pragmatic language competencies (PLC)	People who Stutter	91 items, 2 parts. Part 1 (27 items) covers level of ease using language in different contexts; part 2 (64 items) covers ease of use of 16 PLCs in three categories: Precursors, basic exchanges, and extended literal and non‐literal language.	5‐point scale, scale from 1 = never easy, 5 = always easy. Scores based on part 2 items (three categories and total score).	Portuguese
**Conversation and Communication Questionnaire for people with aphasia (CCQA)**	Horton, S., K. Humby, and J.‐C. Herold. 2020. “Development and Preliminary Validation of a Patient‐Reported Outcome Measure for Conversation Partner Schemes: The CONVERSATION and Communication Questionnaire for People with Aphasia (CCQA).” *Aphasiology* 34, no. 9: 1112–1137.	Participation in conversation (focus on evaluation of conversation partner schemes)	People with aphasia	14 items covering 4 factors: Realising opportunities for participation in conversation, feelings about speech, actions and attitudes of others, experience of participation in conversations.	4‐point scale, 0 = strongly agree, 3 = strongly disagree.	English
**Communication Confidence Rating Scale in aphasia (CCRSA)**	Babbitt, E. M., and L. R. Cherney. 2010. “Communication Confidence in Persons with Aphasia.” *Topics in Stroke Rehabilitation* 17, no. 3: 214–223.	Confidence in communication	People with aphasia	10 items	Rating scale rating 0–100	
**Communication Outcome After Stroke (COAST)**	Long A., A. Hesketh, G. Paszek, M. Booth, and A. Bowen. 2008. “Development of a Reliable Self‐Report Outcome Measure for Pragmatic Trials of Communication Therapy Following Stroke: The Communication Outcome after Stroke (COAST) Scale.” *Clinical Rehabilitation* 22, no. 12: 1083–1094.	Communication effectiveness and impact of communication on quality of life	People with aphasia	20 items: 15 on communication effectiveness and 5 on impact of communication on quality of life.	5‐point scale, 0–4. Scores are converted to T‐scores with mean = 50, SD = 10.	English
**Communicative Activities Checklist (COMACT)**	Aujla, S., M. Cruice, N. Botting, L. Worrall, and L. Hickson. 2016. “Preliminary Psychometric Analyses of Two Assessment Measures Quantifying Communicative and Social Activities: the COMACT and SOCACT.” *Aphasiology* 30, no. 8: 898–921.	Communication activities	People with aphasia	45 items, covers talking, listening, reading and writing.	1 point for each activity engaged in. Max score = 45. Frequency of participation also reported	English
**Communicative Participation Item Bank (CPIB)**	Yorkston, K. M., C. R. Baylor, J. Dietz, B. J. Dudgeon, T. Eadie, R. M. Miller, and D. Amtmann. 2008. “Developing a Scale of Communicative Participation: a Cognitive Interviewing Study.” *Disability and Rehabilitation* 30, no. 6: 425–433.	Communicative participation	Generic—people with speech, voice, language and communication disorders	Item bank of 46 items, with adaptive and shorter 10 item forms.	0–3 four point scale going from ‘not at all’ to ‘very much’	English
**Dysarthria Impact Scale (DIP)**	Walshe, M., R. K. Peach, and N. Miller. 2009. “Dysarthria Impact Profile: Development of a Scale to Measure Psychosocial Effects.” *International Journal of Language & Communication Disorders* 44, no. 5: 693–715.	Psychosocial impact (i.e., the psychological and social consequences) of dysarthria that can directly affect participation	People with acquired dysarthria due to a range of different aetiologies	48 statements covering 5 sections. A: Effect of dysarthria on the speaker. B: Acceptance of dysarthria. C: Perception of others’ reaction to speech. D: How dysarthria affects communication with others. E: Dysarthria relative to other worries and concerns	Response options: 1–5 strongly agree to strongly disagree scale for sections A‐D. 1–5 most to least concerned for section E. Scores range 1–5 with 1 = most negative to 5 = least negative.	English
**Experienced Communication in Dementia Questionnaire (ECD‐P)**	Olthof‐Nefkens, M. W., E. W. Derksen, B. J. de Swart, M. W. Nijhuis‐van der Sanden, and J. G. Kalf. 2021. “Development of the Experienced Communication in Dementia Questionnaire: A Qualitative Study.” *INQUIRY: The Journal of Health Care Organization, Provision, and Financing* 58: 00469580211028181.	Experienced communication of persons with dementia (ECD‐P) and their caregivers (ECD‐C)	People with dementia and caregivers	ECD‐P: two parts, Part 1 with 22 items and Part 2 with two items (total 24 items). ECD‐C same 24 items from the perspective of the caregiver and an additional third part of 5 items (total 29 items)	Part 1 (and 3 for ECD‐C): 4‐point Likert scales, either for agreement (fully disagree‐fully agree) or for frequency (during every conversation‐every day‐every week‐almost) never). Part 2: 1 (poor) to 10 (excellent). Scores: scores 0–3.	Dutch
**Emotional Communication in Hearing Questionnaire (EMO‐CHeQ)**	Singh, G., L. Liskovoi, S. Launer, and F. Russo. 2019. “The Emotional Communication in Hearing Questionnaire (EMO‐CHeQ): Development and Evaluation.” *Ear and Hearing* 40, no. 2: 260.	Experiences of hearing and handicap when listening to signals that contain emotion information.	Normal hearing/Hearing impairment (with without hearing aids)	16 items covering four subscales: talker characteristics, speech production, listening in complex situations, and socio‐emotional well‐being)	5‐point Likert scale ranging from 1 = strongly disagree to 5 = strongly agree	English
**Freiburg Questionnaire of linguistic pragmatics (FQLP)**	Riedel, A., H. Suh, V. Haser, I. Hermann, D. Ebert, D. Riemann, … and L. P. Hölzel. 2014. “Freiburg Questionnaire of Linguistic Pragmatics (FQLP): Psychometric Properties based on a Psychiatric Sample.” *BMC Psychiatry* 14, no. 1: 1–10.	Perception of pragmatic speech abilities	People with Asperger's syndrome	11 items, one factor	4‐point scale going from 1 = agree to 4 = disagree (two items reverse scoring). Score range 11–44	German
**Health‐related quality of life in Huntington disease (HDQLIFE)**	Carlozzi, N. E., and D. S. Tulsky. 2013. “Identification of Health‐related Quality of Life (HRQOL) Issues Relevant to Individuals with Huntington Disease.” *Journal of Health Psychology* 18, no. 2: 212–225.	Health‐related quality of life	People with Huntington disease	Measurement system comprising a battery of HRQOL measures including speech, swallowing, chorea and end of life concerns. Speech 27 items (short form 6 items), Swallowing 15 items (short form 6 items).	Response options not specified. Scores: summed scores converted to *t*‐scores	English
**Living with Dysarthria (LwD)**	Hartelius, L., M. Elmberg, R. Holm, A. S. Lövberg, and S. Nikolaidis. 2008. “Living with Dysarthria: Evaluation of a Self‐report Questionnaire.” *Folia Phoniatrica et Logopaedica* 60, no. 1: 11‐19.	Perception of self and speech difficulties and adjustment	Neurogenic speech disorders	50 statements divided into 10 sections. Section 1: communication problems related to speech; Section 2: language/cognition and 3 fatigue. Sections 4–6: on effects of emotions, persons, situations on communication. Section 7: restrictions on roles. Section 8: what contributes to communicative changes. Section 9: how communication is affected, (less frequent, increased difficulty or need for assistance from others). Section 10: strategies used to increase communicative function.	Scores: 0–5, 0=definitely false, 5=definitely true	Swedish
**Health‐related quality of life for clinical research in neurology (NeuroQOL)**	Cella, D., J. S. Lai, C. J. Nowinski, D. Victorson, A. Peterman, D. Miller, F. Bethoux, A. Heinemann, S. Rubin, J. E. Cavazos, A. T. Reder, R. Sufit, T. Simuni, G. L. Holmes, A. Siderowf, V. Wojna, R. Bode, N. McKinney, T. Podrabsky, K. Wortman, … C. Moy. 2012. “Neuro‐QOL: Brief Measures of Health‐related Quality of Life for Clinical Research in Neurology.” *Neurology* 78, no. 23: 1860–1867.	Quality of life	Neurological conditions	13 short measures (8–9 items each) of different QOL domains across physical, mental, and social health.	5‐point scale, 1 = never, 5 = always. Scores converted to *t*‐scores with mean = 50, SD = 10	English
**Overall Assessment of the Speaker's Experience of Stuttering (OASES‐A)**	Yaruss, J. S., and R. W. Quesal. 2006. “Overall Assessment of the Speaker's Experience of Stuttering (OASES): Documenting Multiple Outcomes in Stuttering Treatment.” *Journal of Fluency Disorders* 31, no. 2: 90–115.	Experience of stuttering across ICF domains	People who stutter	100 items covering four sections: (a) general perspectives about stuttering, (b) affective, behavioural, and cognitive reactions to stuttering, (c) functional communication difficulties, and (d) of quality of life.	5‐point scale, 1 = always, 2 = never. Scores are converted so that impact (section) scores range 20–100	English
**Quality of life measurement and outcome in aphasia (QLQA)**	Spaccavento, S., A. Craca, M. Del Prete, R. Falcone, A. Colucci, A. Di Palma, and A. Loverre. 2013. “Quality of Life Measurement and Outcome in Aphasia.” *Neuropsychiatric Disease and Treatment* 27–37.	(Impact of language disorder on) Quality of life	People with aphasia	37 items, covering three domains, communication, autonomy, and psychological condition.	5‐point scale, 0= the individual is able to successfully perform the behaviour 0% of the time, 4 = the individual is able to successfully perform the behaviour 100% of the time	Italian
**Quality of life in the dysarthric speaker (QOL‐DYS)**	Piacentini, V., A. Zuin, D. Cattaneo, and A. Schindler. 2011. “Reliability and Validity of an Instrument to Measure Quality of Life in the Dysarthric Speaker.” *Folia phoniatrica et logopaedica: official organ of the International Association of Logopedics and Phoniatrics (IALP)* 63, no. 6: 289–295.	Quality of life	People with dysarthria	40 items. 4 sections with 10 items each. Sections include: ‘Speech Characteristic of the Word’, ‘Situational Difficulty’, ‘Compensatory Strategies’, and ‘Perceived Reactions of Others’.	0–4 five point scale going from ‘never’, ‘almost never’ to ‘always’. The total score ranges between 0 and 160. 0 ‐ optimal QOL,160 ‐ severely compromised QOL.	Italian
**Satisfaction with Communication in Everyday Speaking Situations (SCESS)**	Karimi, H., M. Onslow, M. Jones, S. O'Brian, A. Packman, R. Menzies, S. Reilly, M. Sommer, and S. Jelčić‐Jakšić. 2018. “The Satisfaction with Communication in Everyday Speaking Situations (SCESS) Scale: An Overarching Outcome Measure of Treatment Effect.” *Journal of Fluency Disorders* 58: 77–85.	Communicative Satisfaction (to be used in clinical trials as an outcome measure for stuttering treatment)	People who stutter	I item	0–9 ten point scale ranging from "Extremely Satisfied" to "Extremely Dissatisfied"	English
**Self‐Efficacy for Situational Communication Management Questionnaire (SESMQ)**	*No studies on the PROM development, only psychometrics*	Situational communication management	People with deafness	40 items. 20 situations with 2 items each.	0–10 eleven point scale with two types of response options for each situation, ranging from ‘Not well at all’ to ‘very well’, and ‘Not confident at all’ to ‘very confident’.	English
**Stuttering generalization self‐measure (SGSM)**	Alameer, M., L. Meteyard, and D. Ward. 2017. “Stuttering Generalization Self‐measure: Preliminary Development of a Self‐measuring Tool.” *Journal of Fluency Disorders* 53: 41–51.	Stuttering severity and speech anxiety in real life situations	People who stutter	9 items demanding responses on two categories: Fluency and speech anxiety.	1–9 nine point scale ranging from ‘No Stuttering’ to ‘Severe Stuttering’ for assessing fluency and 1–5 five point scale ranging from ‘No Anxiety’ to ‘Severe Anxiety’ for assessing speech anxiety.	English
**Speech Handicap Index (SHI)**	Rinkel, R. N., I. M. Verdonck‐de Leeuw, E. J. van Reij, N. K. Aaronson, and C. R. Leemans. 2008. “Speech Handicap Index in Patients with Oral and Pharyngeal Cancer: Better Understanding of Patients' Complaints.” *Head & Neck* 30, no. 7: 868–874.	Speech Problems	People with head and neck cancer	30 items. 2 subscales: speech function and psychosocial functioning related to speech.	5‐point scale ranging from ‘never’, ‘almost never’, to ‘always’ for 29 items. One item to assess overall speech quality item with four response categories ‘good’, ‘reasonable’, ‘poor’, and ‘severe’. Score range: 0–120, with higher scores indicating higher levels of speech‐ related problems.	Dutch
**Traumatic Brain Injury‐ Quality of Life (TBI‐QOL) Communication Item Bank**	Cohen, M. L., P. A. Kisala, A. J. Boulton, N. E. Carlozzi, C. V. Cook, and D. S. Tulsky. 2019. “Development and Psychometric Characteristics of the TBI‐QOL Communication Item Bank.” *The Journal of Head Trauma Rehabilitation* 34, no. 5: 326–339.	Quality of life	People with TBI	31 items. Also available as a fixed‐length short form (9 items), and as a computerized adaptive test.	Five point scale used with 2 sets of response options. Set A ranges from 5‐‘none’, 4‐‘a little’, 3‐‘somewhat’, 2‐‘a lot’, 1‐‘cannot do’; Set B ranges from 5‐‘never’, 4‐‘rarely’ (once), 3‐‘sometimes’ (2 or 3 times), 2‐‘often’ (about once a day), 1‐‘very often’ (several times a day). All scores across all forms are converted to *t*‐metrics and are relative to each other.	English
**Verbal Activity Log (VAL)**	Haddad, M. M., E. Taub, G. Uswatte, M. L. Johnson, V. W. Mark, A. Barghi, E. Byrom, X. Zhou, and C. M. Rodriguez. 2017. “Assessing the Amount of Spontaneous Real‐World Spoken Language in Aphasia: Validation of Two Methods.” *American Journal of Speech‐language Pathology* 26, no. 2: 316–326.	Real world speech	People with aphasia	10 items covering a range of situations.	Two 0–5 six point Likert scales. Amount Scale and Quality Scale. Anchors/ Descriptions are provided for each whole number score, e.g., on the Amount Scale, 3‐ ‘Used speech about half as much as before the stroke (half the time) (50%)’. Half point ratings are also allowed	English
**Vocal Fatigue Index (VFI)**	Nanjundeswaran, C., B. H. Jacobson, J. Gartner‐Schmidt, and K. Verdolini Abbott. 2015. “Vocal Fatigue Index (VFI): Development and Validation.” *Journal of Voice: Official Journal of the Voice Foundation* 29, no. 4: 433–440.	Vocal fatigue	People with disorders causing vocal fatigue (irrespective of aetiology)	19 items	0–4 five point scale going from ‘never’, ‘almost never’ to ‘always’.	English

### 2.1 Search

For each scale identified, PROM development and content validity studies were identified by searching the articles identified in the Ter Wal study for relevant references. These papers, in turn, were examined for any further relevant references. The scales and relevant documents (e.g., manuals, theses, and books) were also sought where relevant. Full‐text studies asking clients over 18 years old with communication disorders or professionals (e.g., researchers, clinicians) to generate new or evaluate existing items (scale development) and/or assess the relevance, comprehensiveness, and/or comprehensibility of a scale (content validity) were included. Any type of report (e.g., thesis, book, online content, article) reporting on development and content validity was included. Studies evaluating one or more of the other measurement properties (e.g., COSMIN Box 3: structural validity) were excluded. Studies that used the scales only as outcome measures or for validation of other scales were excluded.

### 2.2 Evaluation of the Measurement Properties of the Included PROMs

Where multiple articles were found describing the development and/or validation of the same PROM, they were grouped together. Two authors (LE and KH) were trained in the COSMIN methodology. Calibration between the authors (AP, AV, LE & KH) was performed by independently rating four scales one after the other and discussing findings, questions, and discrepancies to reach a consensus. Scales chosen for initial analysis were selected at random. After the authors agreed they were sufficiently familiar with the procedure, scales were divided equally. Evaluation ratings were completed by all authors (AP, AV, LE and KH), with every measure rated by two authors independently and then discussed to achieve consensus. The team gathered in the same workspace and spent 3 consecutive days on rating to ensure each measure independently rated was promptly discussed and differences were resolved in a consistent manner. To avoid discrepancies between rater pairs, researchers rotated during the process. AV & LE and KH & AP worked together in pairs to complete 50% of the scale ratings before rotating so that AV & KH, and LE & AP completed 25% of the remaining scales, and AV & AP, and KH and LE completed the last 25%. The process of rating followed the COSMIN Methodology for assessing the content validity of PROMs user manual (Terwee, Prinsen, Chiarotto, de Vet, et al. 2018).

**FIGURE 1:**
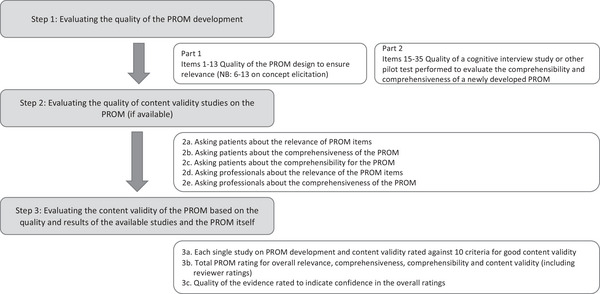
Overview of the three stages in the COSMIN rating process (based on Terwee, Prinsen, Chiarotto, de Vet, et al. 2018).

There were three stages to the evaluation process comprising Step 1: evaluating the quality of the PROM development, Step 2: evaluating the quality of content validity studies on the PROM (if available) and Step 3: evaluating the content validity of the PROM overall, based on the quality and results of the available studies and the PROM itself, using the scoring system presented in the COSMIN Methodology for assessing the content validity of PROMs user manual (Terwee, Prinsen, Chiarotto, de Vet, et al. 2018). Figure 1 provides an overview of the three stages in the rating process.

#### 2.2.1 Step 1

Evaluation of the quality of the PROM development consisted of two parts (COSMIN box 1): outlining 35 standards against which each PROM was rated. Part 1 described 13 standards focused on evaluating the quality of the PROM design to ensure relevance (item generation). A subset of these (6–13) focused on the concept elicitation study. A concept elicitation study should describe a qualitative or mixed‐methods study for identifying relevant items for a new PROM. A standard was rated as very good when there was evidence that the quality aspect of the study to which the standard was referring was adequate. A standard was rated as adequate when relevant information was not reported in an article but it could be assumed that the quality aspect was adequate, for example, saturation could be assumed because a large number of people were interviewed. A standard was rated as doubtful if it was doubtful whether the quality aspect was adequate and as inadequate when evidence was provided that it was not adequate.

Part 2 described standards to evaluate the quality of a cognitive interview study or other pilot test performed to evaluate the comprehensibility and comprehensiveness of a newly developed PROM. Twenty standards (items 15–35) were rated against the same criteria described above (very good, adequate, doubtful, inadequate). If a cognitive interview study or any other kind of pilot test was not performed, the rest of the box was skipped, and the total quality of the PROM development study was rated as inadequate. An overall rating of PROM development was obtained by taking the lowest rating of all these standards. The COSMIN guidance justifies the ‘worst score counts’ method because good methodological aspects of a study cannot compensate for the poor aspects.

#### 2.2.2 Step 2

Evaluation of the quality of content validity studies on the PROM (COSMIN box 2) consisted of 31 standards across five parts: (2a) Asking patients about the relevance of the PROM items. (2b) Asking patients about the comprehensiveness of the PROM. (2c) Asking patients about the comprehensibility of the PROM. (2d) Asking professionals about the relevance of the PROM items. (2e) Asking professionals about the comprehensiveness of the PROM. The five parts outline the standards for collecting data to support content validity by conducting qualitative research such as focus groups or individual interviews to capture the perspectives of patients and professionals on issues of importance relative to each item on the PROM. These standards were rated against the same criteria described above in Step 1 (very good, adequate, doubtful, inadequate).

#### 2.2.3 Step 3

In this step, the content validity of the PROM was rated based on a summary of all available evidence on the PROM development and content validity studies. Additionally, though weighted less, a reviewer rating was completed. However, as per COSMIN guidance, if there were no content validity studies or only content validity studies of inadequate quality, and the PROM development was of inadequate quality, then the rating of the reviewers determined the overall ratings. In this study, the authors, who are all qualified SLTs with expertise in adult communication disorders, acted as reviewers.

**FIGURE 2:**
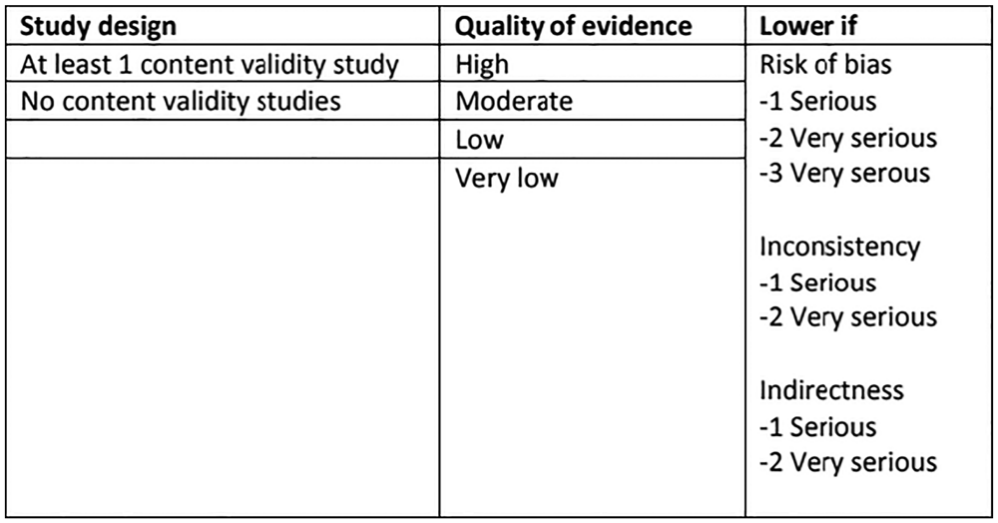
Grading the quality of evidence on content validity (replicated with permission from Terwee, Prinsen, Chiarotto, de Vet, et al. 2018, 62).

First (Step 3a), each single study on PROM development and content validity was rated against 10 criteria for good content validity using a rating scale of sufficient (+) meaning > or = to 85% of the items on the PROM fulfil the criteria, insufficient (−) when < 85% of the items on PROM fulfil the criteria or indeterminate (?) when not enough information was available or the quality was inadequate. Having rated each single study and collected reviewer ratings on each PROM in this way, a total PROM rating was completed (Stage 3b) to determine overall relevance, comprehensiveness, comprehensibility and content validity. These overall PROM ratings were rated as sufficient (+), insufficient (−), or inconsistent (±). Finally (Stage 3c), the quality of the evidence was rated to indicate our confidence in the overall ratings. The evidence was rated as high, moderate, low, or very low, starting with high, where there was at least one content validity study, or moderate, where there was no content validity study, and lowering the rating for risk of bias, inconsistency, and indirectness, following a modified GRADE approach; see Figure 2.

## 3 Results

A total of 34 full‐text articles were reviewed in relation to the 25 included PROM communication scales. Thirty of these articles were on PROM development. For four scales, there were at least two papers describing (parts of) PROM development: CPIB, HDQLIFE, NeuroQOL and TBI‐QOL. These four scales are all item banks. Assessment of content validity beyond scale development was reported for only five scales: the ACOM, CCQA, COAST, CPIB and ECD‐P.

In terms of target population, 8 of the 25 scales were developed for people with aphasia, four for people who stutter, three for people with dysarthria, two for people who are deaf or hard of hearing, and one each for people with dementia, Huntington's disease, head and neck cancer, traumatic brain injury, Asperger's Syndrome, and vocal fatigue. Two were generic PROMs: one for people with neurological conditions (Neuro‐Q0L), and one for people with speech, language, and voice problems (CPIB). In the included studies, the NeuroQOL was tested with people with Alzheimer disease, multiple sclerosis (MS), amyotrophic lateral sclerosis syndrome (ALS), Parkinson disease, stroke (unclear if people with aphasia were included), and adult and paediatric epilepsy conditions; and carers of people with Alzheimer's disease, stroke, and paediatric epilepsy. The CPIB was tested with people with spasmodic dysphonia, MS, stroke (inc., aphasia, apraxia of speech, dysarthria), stuttering, Parkinson's disease, ALS, and laryngectomy.

### 3.1 Step 1: Evaluating the Quality of PROM Development

Table 2 shows that the quality of PROM development was rated as inadequate for 21 of 25 PROMs and as doubtful for four PROMs. For the PROM‐design phase, two scales were rated as adequate (NeuroQOL and TBI‐QoL). However, as cognitive interview studies performed were rated as doubtful (NeuroQOL) and inadequate (TBI‐QoL), the final ratings were doubtful and inadequate, respectively. For all other scales, the PROM‐design phase was rated as inadequate or doubtful. Cognitive interview studies were performed for around half of the identified PROMs and were rated as doubtful or inadequate for all scales. In terms of the cognitive interviewing for people with aphasia, studies noted that extensive support was provided. For example, ‘face‐to‐face interview assisted survey groups’ with people with aphasia were used to assess candidate items during the development of ACOM (Doyle et al. 2008).

**TABLE 2:** Summary of review PROM Development studies.

Reference to the paper(s) in which the PROM development studies are reported.	PROM	Language in which the PROM was developed	PROM design							Cognitive interview (CI) study^2^			Total PROM development
			General design requirements					Concept elicitation^1^	Total PROM design	General design requirements	Comprehensibility	Total CI study	
			Clear construct	Clear origin of construct	Clear target population	Clear context of use	Developed in target population			Performed in sample representing the target population			
Doyle, P. J., M. R. McNeil, K. Le, W. D. Hula, and M. B. Ventura. 2008. “Measuring Communicative Functioning in Community‐Dwelling Stroke Survivors: Conceptual Foundation and Item Development.” *Aphasiology* 22, no. 7–8: 718–728.	ACOM	English	V	V	V	A	V	I	I	V	D	D	**I**
Swinburn, K., W. Best, S. Beeke, M. Cruice, L. Smith, E. P. Willis, K. Ledingham, J. Sweeney, and S. J. McVicker. 2018. “A Concise Patient Reported Outcome Measure for People with Aphasia: The Aphasia Impact Questionnaire 21.” *Aphasiology*	AIQ	English	I	D	I	V	A	I	I				**I**
Valente, A. R. S., A. Hall, H. Alvelos, M. Leahy, and L. M. T. Jesus. 2019. “Reliability and Validity Evidence of the Assessment of Language Use in Social Contexts for Adults (ALUSCA).” *Logopedics, Phoniatrics, Vocology* 44, no. 4: 166–177.	ALUSCA	English	V	V	I	A	I	I	I	D		I	**I**
Horton, S., K. Humby, and C. Jerosch‐Herold. 2020. “Development and Preliminary Validation of a Patient‐reported Outcome Measure for Conversation Partner Schemes: The Conversation and Communication Questionnaire for People with Aphasia (CCQA).” *Aphasiology* 34, no. 9: 1112–1137.	CCQA	English	I	V	V	V	V	D	D				**I**
Babbitt, E. M., and L. R. Cherney. 2010. “Communication Confidence in Persons with Aphasia.” *Topics in Stroke Rehabilitation* 17, no. 3: 214–223.	CCRSA	English	V	V	V	D	I		I				**I**
Long, A., A. Hesketh, G. Paszek, M. Booth, and A. Bowen. 2008. “Development of a Reliable Self‐report Outcome Measure for Pragmatic Trials of Communication Therapy following Stroke: The Communication Outcome after Stroke (COAST) Scale.” *Clinical Rehabilitation* 22, no. 12: 1083–1094.	COAST	English	A	V	V	V	D	I	I	V	I	I	**I**
Aujla, S., N. Botting, L. Worrall, L. Hickson, and M. Cruice. 2016. “Preliminary Psychometric Analyses of Two Assessment Measures Quantifying Communicative and Social Activities: The COMACT and SOCACT.” *Aphasiology* 30, no. 8: 898–921.	COMACT	English	I	V	V	V	I						**I**
Yorkston, K. M., C. R. Baylor, E. R. Klasner, J. Deitz, B. J. Dudgeon, T. Eadie, R. M. Miller, and D. Amtmann. 2007. “Satisfaction with Communicative Participation as Defined by Adults with Multiple Sclerosis: A Qualitative Study.” *Journal of Communication Disorders* 40, no. 6: 433–451.	CPIB ‐	English	V	V	N	D	I	I	I				**D**
Yorkston, K. M., C. R. Baylor, J. Dietz, B. J. Dudgeon, T. Eadie, R. M. Miller, and D. Amtmann. 2008. “Developing a Scale of Communicative Participation: A Cognitive Interviewing Study.” Disability and Rehabilitation 30, no. 6: 425–433.		N/A	V	V	D	D	I			D	D	I	
Baylor, C., M. Burns, T. Eadie, D. Britton, and K. Yorkston. 2011. “A Qualitative Study of Interference with Communicative Participation Across Communication Disorders in Adults.” *American Journal of Speech‐language Pathology* 20, no. 4: 269–287.	—	N/A	V	V	V	V	V			V	D	D	
Walshe, M., R. K. Peach, and N. Miller. 2009. “Dysarthria Impact Profile: Development of a Scale to Measure Psychosocial Effects.” *International Journal of Language & Communication Disorders* 44, no. 5: 693–715.	DIP	English	V	V	V	V	D	I	I	D	D	I	**I**
Olthof‐Nefkens, M. W. L. J., E. W. Derksen C., B. J. M. de Swart, M. W. G. Nijhuis‐van der Sanden, and J. G. Kalf. 2021. “Development of the Experienced Communication in Dementia Questionnaire: A Qualitative Study.” *Inquiry: A Journal of Medical Care Organization, Provision and Financing* 58: 469580211028181.	ECD‐P	Dutch	V	V	V	V	D	I	I	V	D	D	**I**
Singh, G., L. Liskovoi, S. Launer, and F. Russo. 2019. “The Emotional Communication in Hearing Questionnaire (EMO‐CHeQ): Development and Evaluation.” *Ear and Hearing* 40, no. 2: 260–271.	EMO‐CHeQ	English	V	V	D	D	I		I	D	I	I	**I**
Hermann, I., V. Haser, L.T. van Elst, et al. 2013. “Automatic Metaphor Processing in Adults with Asperger Syndrome: A Metaphor Interference Effect Task.” *Eur Arch Psychiatry Clin Neurosci* 263, no. Suppl 2: 177–187.	FQLP	German	V	V	V	D	A	I	I				**I**
Carlozzi, N. E., and D. S. Tulsky. 2013. “Identification of Health‐Related Quality of Life (HRQOL) Issues Relevant to Individuals with Huntington Disease.” *Journal of Health Psychology* 18, no. 2: 212–225.	HDQLIFE	English	V	V	V	V	V	D	D				**D**
Carlozzi, N. E., S. G. Schilling, J. S. Lai, J. S. Paulsen, E. A. Hahn, J. S. Perlmutter, C. A. Ross, and D. Cella. 2016. “HDQLIFE: Development and Assessment of Health‐Related Quality of Life in Huntington Disease (HD).” *Quality of Life Research: An International Journal of Quality of Life Aspects of Treatment, Care and Rehabilitation* 25, no. 10: 2441–2455.	—	N/A								V	D	D	
Hartelius, L., M. Elmberg, R. Holm, A. S. Lovberg, and S. Nikolaidis. 2008. “Living with Dysarthria: Evaluation of a Self‐Report Questionnaire.” *Folia phoniatrica et logopaedica: Official Organ of the International Association of Logopedics and Phoniatrics (IALP)* 60, no. 1: 11–19.	LwD	Swedish	V	V	V	D	I		I				**I**
Perez, L., J. Huang, L. Jansky, C. Nowinski, D. Victorson, A. Peterman, and D. Cella. 2007. “Using Focus Groups to Inform the Neuro‐QOL Measurement Tool: Exploring Patient‐Centered, Health‐Related Quality of Life Concepts Across Neurological Conditions.” *The Journal of Neuroscience Nursing: Journal of the American Association of Neuroscience Nurses* 39, no. 6: 342–353.	NeuroQOL	English	V	V	V	V	A	V	A				**D**
Lynch, E. B., Z. Butt, A. Heinemann, D. Victorson, C. J. Nowinski, L. Perez, and D. Cella. 2008. “A Qualitative Study of Quality of Life After Stroke: The Importance of Social Relationships.” *Journal of Rehabilitation Medicine* 40, no. 7: 518–523.	—	N/A	V	D	V	D	D	I	I				
Cella, D., J. S. Lai, C. J. Nowinski, D. Victorson, A. Peterman, D. Miller, F. Bethoux, A. Heinemann, S. Rubin, J. E. Cavazos, A. T. Reder, R. Sufit, T. Simuni, G. L. Holmes, A. Siderowf, V. Wojna, R. Bode, N. McKinney, T. Podrabsky, K. Wortman, … C. Moy. 2012. “Neuro‐QOL: Brief Measures of Health‐Related Quality of Life for Clinical Research in Eurology.” *Neurology* 78, no. 23: 1860–1867.	—	N/A								V	D	D	
Yaruss, J. S., and R. W. Quesal. 2006. “Overall Assessment of the Speaker's Experience of Stuttering (OASES): Documenting Multiple Outcomes in Stuttering Treatment.” *Journal of Fluency Disorders* 31, no. 2: 90–115.	OASES‐A	English	V	V	I	V	D	I	I	D			**I**
Spaccavento, S., A. Craca, M. Del Prete, R. Falcone, A. Colucci, A. Di Palma, and A. Loverre. 2014. “Quality of Life Measurement and Outcome in Aphasia.” *Neuropsychiatric Disease and Treatment* 10: 27–37.	QLQA	Italian	I	D	V	D	I	I	I				**I**
Piacentini, V., A. Zuin, D. Cattaneo, and A. Schindler. 2011. “Reliability and Validity of an Instrument to Measure Quality of Life in the Dysarthric Speaker.” *Folia phoniatrica et logopaedica: Official Organ of the International Association of Logopedics and Phoniatrics (IALP)* 63, no. 6: 289–295.	QOL‐DYS	Italian	V	D	V	I	A	I	I	V	D	D	**I**
Karimi, H., M. Onslow, M. Jones, S. O'Brian, A. Packman, R. Menzies, S. Reilly, M. Sommer, and S. Jelčić‐Jakšić. 2018. “The Satisfaction with Communication in Everyday Speaking Situations (SCESS) Scale: An Overarching Outcome Measure of Treatment Effect.” *Journal of Fluency Disorders* 58: 77–85.	SCESS	English	V	V	V	V	A	D	D	V	D	D	**D**
*no studies on the PROM development, only psychometrics*	SESMQ	English	—	—	—	—	—	—	—	—	—	—	—
Alameer, M., L. Meteyard, and D. Ward. 2017. “Stuttering Generalization Self‐measure: Preliminary Development of a Self‐measuring Tool.” *Journal of Fluency Disorders* 53: 41–51.	SGSM	English	V	V	V	V	I	I	I				**I**
Rinkel, R. N., I. M. Verdonck‐de Leeuw, E. J. van Reij, N. K. Aaronson, and C. R. Leemans. 2008. “Speech Handicap Index in Patients with Oral and Pharyngeal Cancer: Better Understanding of Patients' Complaints.” *Head & Neck* 30, no. 7: 868–874.	SHI	Dutch	I	I	D	I	I	I	I				**I**
Cohen, M. L., P. A. Kisala, A. J. Boulton, N. E. Carlozzi, C. V. Cook, and D. S. Tulsky. 2019. “Development and Psychometric Characteristics of the TBI‐QOL Communication Item Bank.” *The Journal of Head Trauma Rehabilitation* 34, no. 5: 326–339.	TBI‐QOL	English	V	V	V	V	V	V		D	I	I	**I**
Carlozzi, N. E., D. S. Tulsky, and P. A. Kisala. 2011. “Traumatic Brain Injury Patient‐Reported Outcome Measure: Identification of Health‐Related Quality‐of‐Life Issues Relevant to Individuals with Traumatic Brain Injury.” *Archives of Physical Medicine and Rehabilitation* 92, no. 10 Suppl: S52–S60.		N/A	V	V	V	V	V	A	A				
Haddad, M. M., E. Taub, G. Uswatte, M. L. Johnson, V. W. Mark, A. Barghi, E. Byrom, X. Zhou, and C. M. Rodriguez. 2017. “Assessing the Amount of Spontaneous Real‐World Spoken Language in Aphasia: Validation of Two Methods.” *American Journal of Speech‐Language Pathology* 26, no. 2: 316–326.	VAL	English	V	V	I	V	I	I	I				**I**
Nanjundeswaran, C., B. H. Jacobson, J. Gartner‐Schmidt, and K. Verdolini Abbott. 2015. “Vocal Fatigue Index (VFI): Development and Validation.” *Journal of Voice: Official Journal of the Voice Foundation* 29, no. 4: 433–440.	VFI	English	V	V	V	V	I		I				**I**
V = very good													
A = adequate													
D = doubtful													
I = inadequate													
NA = not applicable													

^1^ When the PROM was not developed in a sample representing the target population, the concept elicitation was not further rated.

^2^ Empty cells indicate that a CI study (or part of it) was not performed.

### 3.2 Step 2 Evaluating the Quality of the Content Validity Studies

Content validity studies were performed for 5 of 25 PROMs included in this review, as can be seen in Table 3. All these studies were rated doubtful or inadequate. A total of five articles were identified, which comprised studies involving patients (*n* = 5), communication partners (*n* = 2) and professionals (*n* = 3). Of the studies involving patients, only one was rated as of adequate quality, three as doubtful quality, and one as inadequate quality. CPIB was the only PROM for which a study aimed to assess both relevance and comprehensibility by way of 1:1 interviews with people with communication difficulties (see Miller et al. 2017). However, it should be noted that while CPIB is designed to be a cross condition measure, the content validity study involved only people with hearing loss (PwHL). The three studies involving healthcare professionals were all of doubtful quality.

**TABLE 3:** Summary of review PROM content validity studies.

Reference to the paper(s) in which the PROM content validity studies are reported.	PROM	Asking patients			Asking experts	
		Relevance	Comprehensiveness	Comprehensibility	Relevance	Comprehensiveness
Doyle, P. J., M. R. McNeil, K. Le, W. D. Hula, and M. B. Ventura. 2008. “Measuring Communicative Functioning in Community‐Dwelling Stroke Survivors: Conceptual Foundation and Item Development.” *Aphasiology* 22 no. 7–8: 718–728.	ACOM	D	D	D	D	D
Horton, S., K. Humby, and C. Jerosch‐Herold. 2020. “Development and Preliminary Validation of a Patient‐Reported Outcome Measure for Conversation Partner Schemes: The Conversation and Communication Questionnaire for People with Aphasia (CCQA).” *Aphasiology* 34, no. 9: 1112–1137.	CCQA	D	I	D	I	I
Long, A., A. Hesketh, G. Paszek, M. Booth, and A. Bowen. 2008. “Development of a Reliable Self‐Report Outcome Measure for Pragmatic Trials of Communication Therapy Following Stroke: The Communication Outcome after Stroke (COAST) Scale.” *Clinical Rehabilitation* 22, no. 12: 1083–1094.	COAST	I	I	I	I	I
Miller, C. W., C. R. Baylor, K. Birch, and K. M. Yorkston. 2017. “Exploring the Relevance of Items in the Communicative Participation Item Bank (CPIB) for Individuals With Hearing Loss.” *American Journal of Audiology* 26, no. 1: 27–37.	CPIB	A	A	A	D	D
Olthof‐Nefkens, M. W., E. W. Derksen, de Swart, B. J., M. W. Nijhuis‐van der Sanden, and J. G. Kalf. 2021. “Development of the Experienced Communication in Dementia Questionnaire: A Qualitative Study.” *INQUIRY: The Journal of Health Care Organization, Provision and Financing* 58: 469580211028181.	ECD‐P	D	I	D	D	D

Abbreviations: A = adequate; D = doubtful; I = inadequate; NA = not applicable; V = very good.

### 3.3 Step 3 Evaluating the Content Validity of the PROM Based on the Quality and Results of the Available Studies and the PROM Itself

Overall, the quality of the available evidence on the content validity of the included PROMs was very low, as can be seen in Table 4. Only a handful of studies were of moderate quality, in particular the studies on the CPIB and the NeuroQOL. In terms of the PROMs’ overall rating, the NeuroQQOL was the only scale rated with a ‘+’ on all four criteria (content validity; relevance, comprehensiveness, and comprehensibility).

**TABLE 4:** Overall rating of the PROMs and quality of the evidence.

	**ACOM**		**AIQ**		**ALUSCA**		**CCQA**		**CCRSA**	
	**Overall rating**	**Quality of evidence**	**Overall rating**	**Quality of evidence**	**Overall rating**	**Quality of evidence**	**Overall rating**	**Quality of evidence**	**Overall rating**	**Quality of evidence**
	**+/−/?**	**High, moderate, low, very low**	**+/−/?**	**High, moderate, low, very low**	**+/−/?**	**High, moderate, low, very low**	**+/−/?**	**High, moderate, low, very low**	**+/−/?**	**High, moderate, low, very low**
Content validity	?	Very low	±	Very low	±	Very low	—	Very low	±	Very low
*Relevance*	?	Very low	+	Very low	±	Very low	—	Very low	±	Very low
*Comprehensiveness*	?	Very low	±	Very low	±	Very low	—	Very low	—	Very low
*Comprehensibility*	?	Very low	±	Very low	±	Very low	—	Very low	±	Very low
	**COAST**		**COMACT**		**CPIB**		**DIP**		**ECD‐P**	
	**Overall rating**	**Quality of evidence**	**Overall rating**	**Quality of evidence**	**Overall rating**	**Quality of evidence**	**Overall rating**	**Quality of evidence**	**Overall rating**	**Quality of evidence**
	**+/−/?**	**High, moderate, low, very low**	**+/−/?**	**High, moderate, low, very low**	**+/−/?**	**High, moderate, low, very low**	**+/−/?**	**High, moderate, low, very low**	**+/−/?**	**High, moderate, low, very low**
Content validity	?	Very low	±	Very low	?	Moderate	?	Very low	?	Very low
*Relevance*	±	Very low	±	Very low	?	Moderate	?	Very low	+/−	Very low
*Comprehensiveness*	?	Very low	±	Very low	+	Moderate	?	Very low	?	Very low
*Comprehensibility*	—	Very low	±	Very low	+/−	Moderate	?	Very low	?	Very low
	**EMO‐CHeQ**		**FQLP**		**HDQOL**		**LWD**		**Neuro‐QoL**	
	**Overall rating**	**Quality of evidence**	**Overall rating**	**Quality of evidence**	**Overall rating**	**Quality of evidence**	**Overall rating**	**Quality of evidence**	**Overall rating**	**Quality of evidence**
	**+/−/?**	**High, moderate, low, very low**	**+/−/?**	**High, moderate, low, very low**	**+/−/?**	**High, moderate, low, very low**	**+/−/?**	**High, moderate, low, very low**	**+/−/?**	**High, moderate, low, very low**
Content validity	±	Very low	—	Very low	?	Low	—	Very low	+	Moderate
*Relevance*	—	Very low	±	Very low	?	Low	—	Very low	+	Moderate
*Comprehensiveness*	+	Very low	—	Very low	+	Moderate	±	Very low	+	Moderate
*Comprehensibility*	±	Very low	±	Very low	?	Low	±	Very low	+	Moderate
	**OASIS**		**QLQA**		**QOL‐DyS**		**SESMQ**		**SCESS**	
	**Overall rating**	**Quality of evidence**	**Overall rating**	**Quality of evidence**	**Overall rating**	**Quality of evidence**	**Overall rating**	**Quality of evidence**	**Overall rating**	**Quality of evidence**
	**+/−/?**	**High, moderate, low, very low**	**+/−/?**	**High, moderate, low, very low**	**+/−/?**	**High, moderate, low, very low**	**+/−/?**	**High, moderate, low, very low**	**+/−/?**	**High, moderate, low, very low**
Content validity	?	Very low	—	Very low	±	Very low	±	Very low	±	Very low
*Relevance*	+	Very low	—	Very low	±	Very low	+	Very low	+	Very low
*Comprehensiveness*	+/−	Very low	—	Very low	—	Very low	?	Very low	?	Very low
*Comprehensibility*	—	Very low	—	Very low	±	Very low	±	Very low	±	Very low
	**SGSM**		**SHI**		**TBI‐QOL**		**VAL**		**VFI**	
	**Overall rating**	**Quality of evidence**	**Overall rating**	**Quality of evidence**	**Overall rating**	**Quality of evidence**	**Overall rating**	**Quality of evidence**	**Overall rating**	**Quality of evidence**
	**+/−/?**	**High, moderate, low, very low**	**+/−/?**	**High, moderate, low, very low**	**+/−/?**	**High, moderate, low, very low**	**+/−/?**	**High, moderate, low, very low**	**+/−/?**	**High, moderate, low, very low**
Content validity	—	Very low	—	Very low	+	Low	—	Very low	—	Very low
*Relevance*	—	Very low	—	Very low	+	Low	—	Very low	—	Very low
*Comprehensiveness*	—	Very low	—	Very low	+	Low	—	Very low	—	Very low
*Comprehensibility*	—	Very low	—	Very low	+	Low	—	Very low	—	Very low

In the absence of good quality studies, the clinician ratings are important. Overall, many of the instruments were considered relevant for the target population. Comprehensiveness and comprehensibility were often more problematic, particularly for the scales for populations that do not typically have comprehension difficulties.

## 4 Discussion

This study explored the content validity of existing PROMs used with adults with communication disorders according to the COSMIN guidelines. Communication disorders included: speech, language, voice, and hearing difficulties. Results of this study highlight the scarcity of high‐quality evidence on the development and content validity of PROMs that aim to capture communication.

Clinimetric and psychometric experts recommend content validity as the first and most important measurement property to consider when selecting a scale. Although this intuitively makes sense, the methodological requirements underpinning this have only recently been agreed on and made explicit (Terwee, Prinsen, Chiarotto, de Vet, et al. 2018). The methodology for evaluating the content validity of PROMs was developed in 2016 in a Delphi study by 158 experts from 21 countries (Terwee, Prinsen, Chiarotto, de Vet, et al. 2018). In 2018, the standards for evaluating the quality of PROM development were published, as well as criteria for what constitutes good content validity of PROMs. These criteria provide transparent and evidence‐based recommendations for the selection of PROMs in systematic reviews to determine whether a PROM is ‘good enough’ to measure outcomes. The scales that were reviewed in our study were all developed prior to the publication of Terwee et al.’s recommendations. Prior to this, the focus of measurement and quality assessment of scales was more on construct validity (e.g., structural validity, hypothesis testing) and reliability.

One of the main limitations identified in the development of the scales under review in this study was that they were often not developed in, or with, a sample of people representing the target population. The construct and target population were often clearly defined, but systematic inclusion of the target population in the development phase was often omitted (or omitted from reporting). Generally, PROMs that were developed specifically for speech and language therapy (often by SLTs) did include some form of patient participation and input. These reports, however, lack detail and methodological rigour. This highlights the future need for both inclusion of the target population in the development stage of a PROM, and the use of rigorous methodology to do so.

Despite this review not providing evidence of a measure that can be defined as ‘best’, the authors identified several measures they rated as having features of ‘good’ development and testing, including the CPIB and NeuroQOL. Interestingly, these scales are both generic, that is, they could be used with different communication disorders. There was no communication condition‐specific measure that met criteria (e.g., aphasia measure or voice measure). Both CPIB and NeuroQOL have been developed as cross‐condition measures that can capture the outcomes for people with communication difficulties arising from a range of reasons. It is possible that studies focused on the development of condition‐specific measures are harder to recruit to, whilst cross‐disorder has more appeal. Alternatively, it may be that in the absence of set criteria like COSMIN, cross‐condition measures that needed to be tested across different conditions to ensure relevance ended up being more rigorously tested, whereas condition‐specific measures were more readily accepted as adequate with limited testing with one group.

PROMs are a promising area for further development of measures as they aim to capture the needs and difficulties experienced by people with communication difficulties. To ensure they achieve this, future studies need to focus on rigorous development of PROMs, specifically testing content validity, prior to any psychometric testing. As a first step, researchers and clinicians should always consider using and, if needed, adapting and further testing existing well‐developed measures. Item banks are particularly suitable for this purpose, as they can be continuously added to and have an established profile, meaning they are both broadly accessible and suitable for comparisons across groups.

In terms of clinical implications, PROMs are useful outcome measures that can provide data demonstrating whether interventions are meeting the needs of the people they are designed for. This study is the first to provide clear information on the relevance, comprehensibility, and comprehensiveness of PROMs to assess communication in adults with communication disorders. By providing the evidence on the quality of content validity of existing communication PROMs, this review can aid decision‐making on outcome measure selection to capture communication outcomes.

One limitation of the study is that only 2 of the 4 reviewers were trained by COSMIN. However, steps were taken to ensure good quality ratings: all the reviewers were experienced clinicians from diverse speech and language research backgrounds, which resulted in a series of rich and insightful discussions; calibration of ratings was performed at the start of the rating process on four measures; and the COSMIN manual was followed closely in all ratings. Other limitations in this area of research included the depth of information provided in the available publications and that many of the studies had been published prior to the introduction of the COSMIN guidance. Future research undertaken in the field of PROM development could usefully employ the COSMIN guidance as a set of principles to inform PROM development as well as study reporting. With its clear guidance on methods to follow and measurement criteria, this will improve the rigour of research conducted and, in turn, reporting. Further, during both the development of new measures and the appraisal of existing ones, explicit consideration should be given to the interpretation of test scores and the inferences that can be made based on them (APA et al. 2014; Iliescu and Greiff 2021).

## 5 Conclusions

The study demonstrates that current PROM tools available for adults with communication disorders do not demonstrate adequate development and content validity. This review has highlighted the future need for both inclusion of the target population in the development stage of a PROM, as well as the use of rigorous methodology to do so. The development of future tools should also focus on expanding currently available item banks rather than generating new tools. Thus, PROMs can influence the development of interventions that demonstrate they meet the needs of people with communication difficulties.

## Data Availability

The data that support the findings of this study are available from the corresponding author upon reasonable request.
